# Genome-wide patterns of genetic variation in sweet and grain sorghum (*Sorghum bicolor*)

**DOI:** 10.1186/gb-2011-12-11-r114

**Published:** 2011-11-21

**Authors:** Lei-Ying Zheng, Xiao-Sen Guo, Bing He, Lian-Jun Sun, Yao Peng, Shan-Shan Dong, Teng-Fei Liu, Shuye Jiang, Srinivasan Ramachandran, Chun-Ming Liu, Hai-Chun Jing

**Affiliations:** 1 Institute of Botany, Chinese Academy of Sciences, Beijing 100093, China; 2 Shenzhen Key Laboratory of Transomics Biotechnologies, BGI-Shenzhen, Shenzhen 518083, China; 3 Temasek Life Sciences Laboratory Limited, 1 Research Link National University of Singapore, Singapore 117604

## Abstract

**Background:**

Sorghum (*Sorghum bicolor*) is globally produced as a source of food, feed, fiber and fuel. Grain and sweet sorghums differ in a number of important traits, including stem sugar and juice accumulation, plant height as well as grain and biomass production. The first whole genome sequence of a grain sorghum is available, but additional genome sequences are required to study genome-wide and intraspecific variation for dissecting the genetic basis of these important traits and for tailor-designed breeding of this important C_4 _crop.

**Results:**

We resequenced two sweet and one grain sorghum inbred lines, and identified a set of nearly 1,500 genes differentiating sweet and grain sorghum. These genes fall into ten major metabolic pathways involved in sugar and starch metabolisms, lignin and coumarin biosynthesis, nucleic acid metabolism, stress responses and DNA damage repair. In addition, we uncovered 1,057,018 SNPs, 99,948 indels of 1 to 10 bp in length and 16,487 presence/absence variations as well as 17,111 copy number variations. The majority of the large-effect SNPs, indels and presence/absence variations resided in the genes containing leucine rich repeats, PPR repeats and disease resistance *R *genes possessing diverse biological functions or under diversifying selection, but were absent in genes that are essential for life.

**Conclusions:**

This is a first report of the identification of genome-wide patterns of genetic variation in sorghum. High-density SNP and indel markers reported here will be a valuable resource for future gene-phenotype studies and the molecular breeding of this important crop and related species.

## Background

Sorghum (*Sorghum bicolor*) originated from Africa and is a pro-poor multipurpose crop providing food, feed, fiber and fuel across a range of agro-ecosystems, especially in those with fragile conditions. Food and Agriculture Organization data show that sorghum is currently the number five most important grain crop and, in the past decade, its yearly production has been stabilized at 60 million tonnes with a harvesting area of 44 million hectares. Sorghum is known as 'the camel amongst crops' and requires much less water than many other cereals and has a remarkable ability to produce a crop under low levels of inputs and adverse stress conditions. Sweet sorghum is a natural variant of common grain sorghum with high stem sugar content and often considered a smart crop because it can produce both food and fuel. As a C_4 _crop with a high level of directly fermentable stem sugars and the ability to produce high biomass under adverse conditions, sweet sorghum is considered an ideal biofuel crop for the first and second generation bioethanol production, particularly having the advantages of exploitation of marginal land and avoiding competing for land for food crops [1-3]. However, the genetic basis for these remarkable traits of sweet sorghum is poorly understood.

Genetic variation consists of sequence variation and structure alteration. Sequence variation normally is manifested by SNPs, short sequence insertions and deletions (indels), microsatellites or simple sequence repeats, and transposable elements. The importance of SNPs and indels was initially realized by the occurrence of human sickle-cell anemia and cystic fibrosis diseases, the dramatic consequences caused by a nucleotide change in the hemoglobin beta gene [4] and a three-base deletion in the gene encoding a cystic fibrosis transmembrane conductance regulator [5-7], respectively. Structural alteration is generally described as presence/absence variations (PAVs) and copy number variations (CNVs), which include large scale deletions, insertions, duplications, inversions and translocations. An effect of CNV on phenotypic variation was documented 75 years ago in *Drosophila melanogaster*, with the Bar eye phenotype being caused by the *Bar *gene duplication [8,9]. In plants, sequence polymorphisms have gained much interest in the academic and breeding communities [10-12]. In several model and crop plants, including *Arabidopsis*, rice and maize, whole genome SNPs and indels have been developed [13-16] for a broad range of functional and evolutionary studies, including association mapping [17,18], genetic diversity [19,20], domestication, and genome evolution [21-23]. The effects of CNVs in plant genomes have only been reported in a few cases. In *Arabidopsis *and rice, array-comparative genome hybridization has been used to examine single-feature polymorphisms [24,25], genomic lesions caused by mutagenesis [26], as well as natural variation [27]. In maize, structural alterations have been reported to violate the intraspecific genome co-linearity [28,29] and contribute to the diversity of a range of important traits, such as heterosis and disease responses [22,30,31]. CNVs also shape the genome diversity of progeny of the immediate next generations in *Arabidopsis *[32]. Nonetheless, CNVs and their importance in plant genome and phenotypic variation are still far from well explored.

*S. bicolor *has three subspecies, namely *arundinaceum, bicolor *and *drummondii*, and the cultivated sorghums are all from *bicolor*, which has five local races, bicolor, caudatum, durra, guinea and kafir [33]. Although sweet sorghum differs phenotypically from grain sorghum and tends to have a sugar-rich juicy stem, taller plant, higher biomass but less grain production [1,34], how sweet sorghum differs genetically from grain sorghum is not well defined [35,36]. Sweet sorghum was found in several local races of *bicolor *subspecies [37], which raises questions about the origin, selection and genetic and genomic basis of sweet sorghum. To address these questions, knowledge about the genome-wide genetic variation between sweet and grain sorghum is required. Such knowledge will also be useful for genetic improvement and tailor-designed breeding of this important crop [38,39]. The availability of the first whole genome sequences for a grain sorghum, BTx623 [40], has provided a template for genome-wide analysis of genetic variation. However, without additional genomes in the same species it is difficult to access hidden genome variation information. We took a next generation sequencing technology and resequenced two sweet and one grain sorghum genomes to identify patterns of sequence polymorphism and structural variation in comparison with the published BTx623 genome. This effort identified a large quantity of SNPs, indels, PAVs and CNVs in sorghum. Comparison of these variation data defined potential genome regions and metabolic pathways associated with sweet- and biofuel-associated traits. The large genome resources provided here are useful for comparative genomics and crop breeding in sorghum and related species.

## Results

### The morphological and physiological characteristics of sorghum lines used for resequencing

Sorghum (*S. bicolor*) accessions Keller, E-Tian, Ji2731 and the reference accession BTx623 were used for this work. Keller is an American-bred elite sweet sorghum line and has been shown to have good performance across a range of environmental conditions [41]. E-Tian (literally meaning Russian Sweet in Chinese) was a sweet sorghum line introduced to China in the early 1970s, while Ji2731 is a representative Chinese *kaoliang *grain sorghum well adapted to the northeast part of China with good seedling establishment and a short growth period.

These sorghum lines differ in a number of agronomic and biofuel-associated traits (Table 1). As expected, the two sweet sorghum lines (Keller and E-Tian) had taller plant height, and higher stem Brix content and stem weight in comparison with the two grain sorghum lines. The Chinese sorghum line Ji2731 had zero accumulation of juice in the stem and the highest grain yield, which are the typical features of Chinese *kaoliang*. The variation in the biological traits in these four sorghum lines provides a basis to study gene-trait associations by examining the sequence polymorphisms and structural variations at the whole-genome level.

**Table 1:** Agronomic and biofuel-associated traits of the sorghum lines used for resequencing

Sorghum line	BTx623	Ji2731	Keller	E-Tian
Plant height (cm)	136.3 ± 9.7	235.6 ± 17.1	381.4 + 26.2	268.0 ± 12.5
Brix (%)	12.2 ± 1.2	0	17.5 + 2.5	15.4 + 2.1
Stem weight (g)	165.0 ± 35.4	252.0 + 42.4	635.0 + 84.9	457.2 + 166.5
Stem diameter (cm)	1.5 ± 0.2	1.5 ± 0.1	1.6 + 0.2	1.4 ± 0.3
Internode number	8.2 ± 0.4	10.7 ± 0.5	13.0 + 0.7	10.9 ± 0.3
Leaf weight (g)	73.4 ± 23.5	70.8 ± 10.9	165.0 + 24.7	79.6 ± 22.1
Panicle length (cm)	26.7 ± 2.8	18.1 ± 1.1	24.2 + 1.8	22.9 ± 2.0
Panicle weight (g)	83.0 ± 14.1	98.4 ± 10.5	58 + 10.6	88.0 + 32.0
Peduncle length (cm)	41.3 ± 2.9	21.1 ± 3.7	56.7 + 3.4	39.9 ± 1.9

The data are shown as mean with standard errors and were collected from plants grown in an experimental field in Gongzhulin, Jilin in three consecutive years from 2007 to 2009.

### Short-read resequencing and landscape of genome variation

A whole-genome shotgun strategy and Illumina Genome Analyser sequencing technology were employed. The genome size of the reference genome BTx623 is 738,787,382, of which the effective size is 697,579,688 (excluding the N bases). We estimated that a 10 × genome coverage should be sufficient for aligning most of the sequences. Nine paired-end sequencing libraries with an insert size around 500 bp, three for each sorghum line, were constructed using DNA samples from 10-day-old etiolated seedlings. Resequencing yielded 620.72 million 44-bp paired-end reads, which comprised 27.31 Gb of high-quality raw data. Sequence reads were aligned to the reference BTx623 genome using SOAP software v2.21 [42]. In total, we achieved an effective depth of ×36.51 coverage, with an average of ×12.17 for each line (Table S1 in Additional file 1).

**Figure 1:**
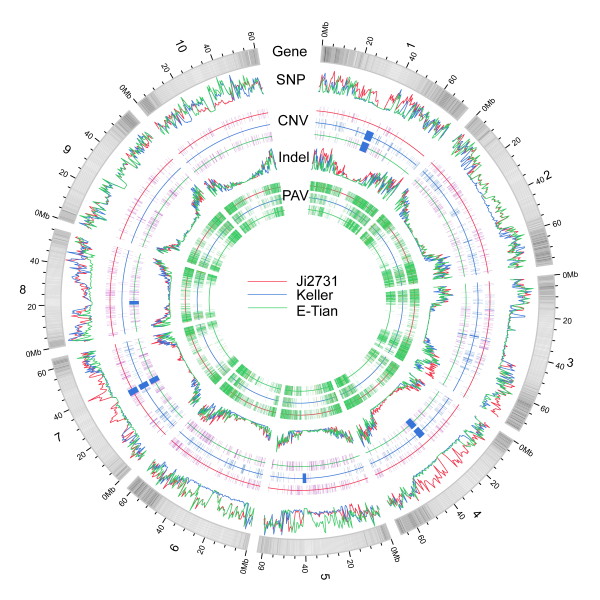
**Genome-wide landscape of genetic variation in *Sorghum bicolor***. Gene density of chromosomes is visualized by line darkness; the more genes on a chromosome region, the darker the color. The purple and blue colors in the CNV ring represent gain and loss of copy number variation, respectively. For PAVs, the green color stands for the absence of variation, whereas pink for the presence of variation.

With these reads and the information from the reference genome BTx623, including physical sequence alignment and gene models, we identified large quantities of SNPs, indels and PAVs (Figure 1). In total, 1,057,018 SNPs among these sorghum genomes, of which 83,262 SNPs were located in the coding regions, were identified (Table S2 in Additional file 1; Additional file 2). SOAPsnp [43] allows the detection of heterozygosity of SNPs and the results showed that the number of heterozygous SNP sites is less than 25% of all SNP sites over the whole genome or in coding regions of the sorghum genome (Table S3 in Additional file 1). The proportions of genic SNPs identified as coding, intronic, or UTR were 42.3%, 50.2%, and 7.5%, respectively. We also identified 99,948 indels ranging from 1 10 bp in length, of which 2,230 were in coding regions (Table S4 in Additional file 1). The proportions of genic indels identified as coding, intronic, or UTR were 9.7%, 75.7%, and 14.6%, respectively. Moreover, 16,487 PAVs with an average length of 2,394 bp were identified (Table S5 in Additional file 1). Coding regions of 1,416 genes in sorghum genomes were included in these PAVs (Table S6 in Additional file 1). CNV was detected by using read depth of coverage [44]. A total of 17,111 CNVs, including 13,427 gains and 3,684 losses ranging from 2 kb to 48 Mb, were detected (5,994 for Ji2713, 3,603 for Keller and 7,514 for E-Tian).

Sanger sequencing technology was used for targeted gene verification. Primer sequences spanning genomic regions predicted to contain genetic variation were used to amplify genomic DNA templates from the three sorghum lines. In this manner, 215 SNPs in 30 genes were selected and 213 were verified using this method, suggesting a prediction accuracy of over 99% (Additional file 3). Similar accuracy was obtained with 48 indels and 9 CNVs.

Because some newly identified genes might exist beyond the currently assembled BTx623 sorghum genome, we assembled unmapped reads with SOAPdenovo and obtained contigs with a total sequence length of 7.2 Mb. Annotation of these contigs showed 73 putative absent genes with an average length of 409 bp (only coding regions were considered; Table S8 in Additional file 1). A Blast search against *Arabidopsis*, rice and maize genome databases revealed that 33 of these genes showed homology with known proteins (E-value < 1e^-6^).

### SNP annotations and large-effect SNPs

SNPs are small differences but with great impact on the variation of genomes and the biological traits. We therefore looked into the SNP annotations in detail and paid special attention to those in genic regions. For this purpose, the newly sequenced grain sorghum BTx623 genome was used as a reference [40]. Bearing in mind that all genome annotations, including that of sorghum, are imperfect and many factors affect the analysis of effects of SNPs - especially the presence of abundant transposon elements in the sorghum genome, which can be difficult to detect when they are present in low copy numbers, are even expressed, or contain fragments of 'real' genes - we analyzed the effects of SNPs using four different gene categories: *bona fide *genes, low-confidence genes, pseudogenes and transposons. We retrieved gene models of the Btx623 genome from the Phytozome database [45], and verified the gene identities using EST information from PlantGDB and maize gene orthologues from MaizeGDB [46]. In the end, the gene sets included 27,640 *bona fide *genes, 5,197 low-confidence genes, 932 transposons and 727 pseudogenes, respectively. These genes fall into 2,637 Pfam families (Additional file 4).

As shown in Table 2 the non-synonymous-to-synonymous ratios in the *bona fide *gene categories were the smallest, increasing from pseudogenes to transposons to low-confidence genes. It was also found that the *bona fide *Pfam-containing genes had a smaller ratio than those of the low-confidence genes and transposons (Table 3). Clearly, the presence of genes involved in transposon functions and transposases have strong effects on increasing the frequencies of SNPs in the genome and hence increasing the diversity of the genomes. When the non-synonymous-to-synonymous ratios in the corresponding gene categories were compared, it was found that, overall, three out of the four gene categories displayed higher ratios in the coding regions of the genome, except for transposons (Tables 2 and 3), indicating that the Pfam domains possibly have fewer amino acid substitutions. A similar drop in the ratios was also reported for the rice genome [20], but the decrease in our sorghum genomes was smaller, which might be related to the imperfection of the genome annotations and the abundant presence of transposon elements.

**Table 2:** Number and distribution of coding region SNPs in the resequenced sorghum genomes

	*Bona fide *genes			Low confidence genes			Transposons			Pseudogenes			Total
Sample	Non-syn	Syn	Non-syn/ Syn	Non-syn	Syn	Non-syn/ Syn	Non-syn	Syn	Non-syn/ Syn	Non-syn	Syn	Non-syn/ Syn	Non-syn/Syn
Ji2731	23,462	18,710	1.25	3,397	1,830	1.86	853	449	1.90	0	0	0.00	1.32
Keller	14,091	10,346	1.36	2,048	1,016	2.02	606	330	1.84	214	164	1.30	1.43
E-Tian	17,781	14,196	1.25	2,386	1,281	1.86	609	374	1.63	314	222	1.41	1.31
Total	38,261	29,625	1.29	5,981	3,113	1.92	1,601	909	1.76	464	327	1.42	1.36

Syn, synonymous.

**Table 3:** Number and distribution of coding region SNPs in Pfam domain-containing genes in the resequenced sorghum genomes

	*Bona fide *genes			Low confidence genes			Transposons			Pseudogenes			Total
	Non-syn	Syn	Non-syn/ Syn	Non-syn	Syn	Non-syn/ Syn	Non-syn	Syn	Non-syn/ Syn	Non-syn	Syn	Non-syn/ Syn	Non-syn/Syn
Ji2731	17,694	14,798	1.20	921	567	1.62	489	259	1.89	0	0	0.00	1.22
Keller	10,503	8,156	1.29	560	295	1.90	301	173	1.74	101	97	1.04	1.31
E-Tian	13,439	11,197	1.20	600	373	1.61	341	219	1.56	146	119	1.23	1.22
Total	28,551	23,182	1.23	1,608	965	1.67	897	504	1.78	209	174	1.20	1.26

Syn, synonymous.

**Figure 2:**
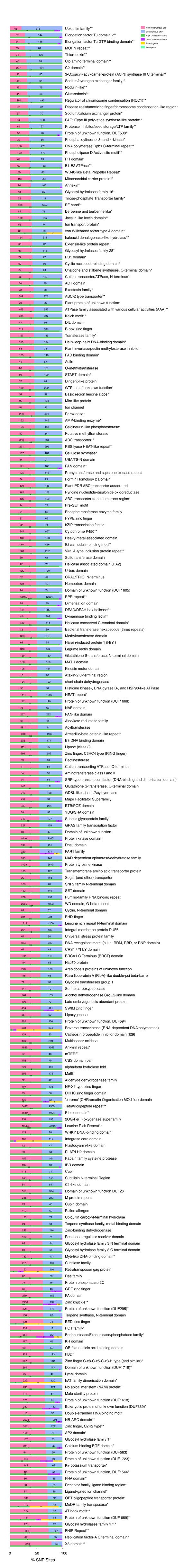
**Number and distribution of non-synonymous and synonymous SNPs in different Pfam genes in the resequenced sorghum genomes**. The Pfam gene families with 30 or more non-synonymous and synonymous SNPs were analyzed and are listed. The Pfam genes are arranged according to the percentages of non-synonymous and synonymous SNP sites. The top Pfam gene families have lower percentages of non-synonymous SNP sites, while the bottom ones have higher percentages of non-synonymous SNP sites. The numbers in the non-synonymous and synonymous horizontal bars show the absolute numbers of SNPs, whereas the numbers in the gene categories are the numbers of genes in each category. For each Pfam, the number of genes in the categories of *bona fide *genes, low-confidence genes, transposons and pseudogenes are also listed. Gene numbers that are lower than 5% of the total genes analyzed are not shown. The chi-square significance of the observed non-synonymous and synonymous SNP distributions for each Pfam group is shown: *P-value < 0.05; **P-value < 0.001.

We further analyzed the distribution of the SNPs in Pfam-containing genes in detail Figure 2. shows the number of non-synonymous and synonymous SNPs in individual Pfam gene families. The number of genes in the four gene categories is also indicated. Nearly half of the SNPs were found in leucine-rich repeats and genes encoding pentatricopeptide repeats (PPRs; Additional file 5), and another 20% of the total SNPs were from 10 Pfam family genes, including genes encoding protein kinases, protein tyrosine kinases, tetratricopeptide repeats, WD domain repeats, zinc knuckles, NB-ARC domains, ankyrin repeats, HEAT repeats, F-boxes, and armadillo/beta-catenin-like repeats. These Pfam family genes often have non-synonymous-to-synonymous ratios higher than 1, making the overall genome ratios high, and when these genes and the transposons were removed for the calculation, the ratio was reduced to close to 1 (data not shown). The finding that sequences encoding leucine-rich repeats and NB-ARC domains had higher ratios of non-synonymous to synonymous SNPs was consistent with findings in *Arabidopsis*, rice and maize, indicative of the diversification of plant disease-resistance proteins caused by pathogen pressure [19,20,22], whereas genes coding for ubiquitin, elongation factor Tu domain 2 and GTP binding domain proteins, all of which have important biological functions essential for life, had the lowest non-synonymous to synonymous ratios. Furthermore, we found that X8 domain and glycosyl hydrolase family 17 containing genes were amongst the families with the highest non-synonymous to synonymous ratios. As controls, the low-confidence genes, transposons and pseudogenes containing various Pfam domains were also included in the analysis. These genes tended to have more non-synonymous SNPs than synonymous SNPs, as represented by genes encoding BED zinc fingers, hAT family dimerization domains, and DUF domains, and MuDR family transposases. As these genes are not normally functional, caution should be exercised when interpreting the gene families with exceptionally high non-synonymous to synonymous ratios, which might not be *bona fide *genes, although evaluation of sorghum Pfam genes using the PlantGDB database showed that 71% of them had EST support.

**Figure 3:**
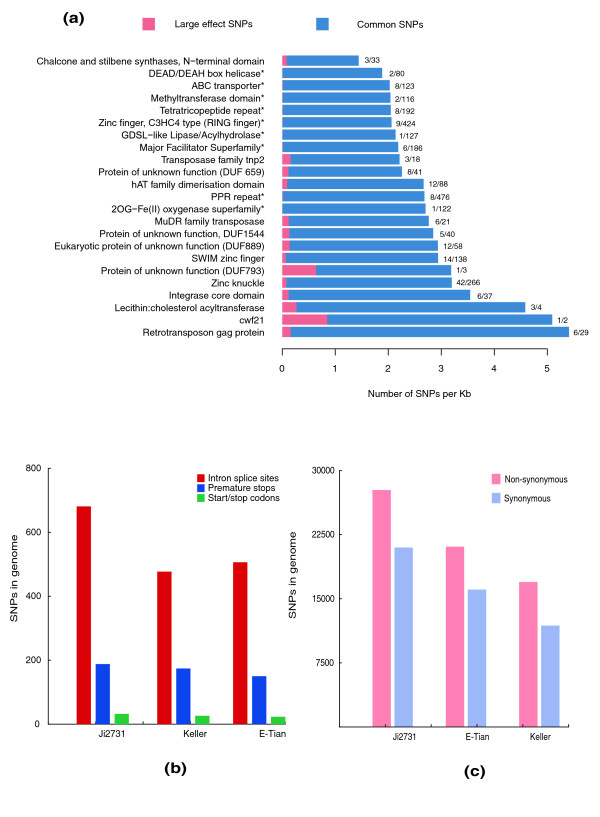
**Annotation and distribution of SNPs**. **(a) **Pfam gene families significantly (*P*-value < 0.01) enriched or depleted with large-effect SNPs. Asterisks indicate Pfam families statistically significantly depleted of large-effect SNPs. **(b) **Statistics of different types of large-effect SNPs. **(c) **Statistics of synonymous and non-synonymous SNPs.

We went further to analyze the distribution of so-called large-effect SNPs, which are predicted to have a potentially disabling effect on gene function. It was found that 1,664 SNPs were expected to induce premature stop codons, 65 to alter initiation methionine residues, 512 to disrupt splicing donor or acceptor sites, and an additional 16 to remove the annotated stop codons, resulting in longer open reading frames (Figure 3b). These large-effect SNPs are statistically significantly (*P*-value < 0.01) enriched in 14 Pfam families and depleted in 9 Pfam families (Figure 3a). However, large-effect SNPs were mostly enriched in transposase genes, such as those encoding MuDR family transposases, Transposase family tnp2 and retrotransposon gag proteins, or genes affected by transposon elements, such as hAT family dimerization domain and DUF domain genes. As these genes do not appear to be functional, we need to experimentally verify the importance of such enrichment. In contrast, those families devoid of large-effect SNPs, including ABC transporter and methyltransferase domain genes, are important for organism survival.

Taken together, we have identified large sets of SNPs, some of which are useful for further downstream functional genomics analyses if the SNPs reside in *bona fide *genes (SNPs residing in non-functional genes or transposon elements can also be useful as molecular markers). However, these results should be viewed with caution at this stage. As the identification of large-effect SNPs depends on the annotation of gene models, the exact number and spectrum of such SNPs will probably be modified when the genome annotation is updated. Furthermore, the sorghum lines used for genome resequencing differ in many traits, and the results may be biased towards having more large-effect SNPs. It would be interesting to include sorghum lines that closely phenotypically resemble each other for comparison. Finally, we analyzed only a limited number of sorghum lines; a representative collection of sorghum lines is required to justify the results obtained.

### Effects of indels and presence/absence variations

**Figure 4:**
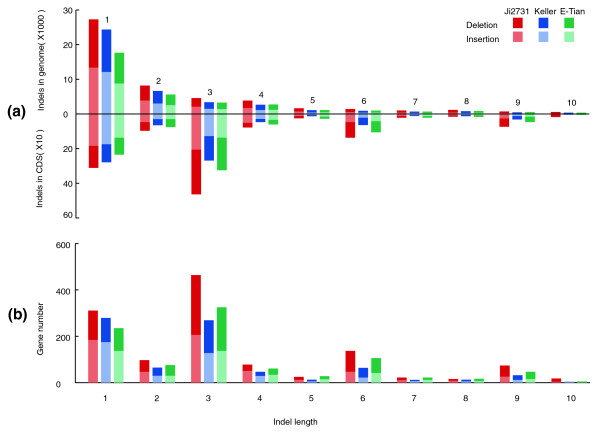
**Distribution of 1- to 10-bp indels in the sorghum genome**. **(a) **Number of indels of different length in the coding sequence (CDS) regions and the whole genome. **(b) **Number of genes that contain indels of different lengths. The figure reveals that 3-bp indels in CDS regions and genes that contain 3-bp indels are of the largest quantity. This implies that 3-bp indels cause the least negative effects on sorghum survival.

We examined the genome-wide patterns of the 1- to 10-bp indels. With increasing indel size, the number of indels decreased. However, our result show that indels that are not multiples of 3 bp and produce frameshift mutations are particularly uncommon in coding regions but relatively common in non-coding regions (Figure 4a). We also found that genes with multiples of 3-bp indels were more commonly present in the genome than those with indels of other lengths (Figure 4b).

We further analyzed the Pfam domains affected by indels. Similar to the situation of non-synonymous SNPs, indels were statistically significantly (*P*-value < 0.001) enriched in NB-ARC and leucine rich repeat domain genes (Table S11 in Additional file 1). Indels were also found enriched in F-box, protein kinase and tyrosine kinase gene families, which are known to possess diverse functions and are suspected to follow a rapid birth-death cycle [47-49]. Although in theory the effects of frame-shifting (1-, 2-, 4-, 5-, 7-, 8- and 10-bp) indels are different from those of non-frame-shift (3-, 6- and 9-bp) ones, we found that, in sorghum, the gene families affected by them were very similar (Additional file 6). PAVs were highly enriched (*P*-value < 0.001) in nine gene families, some of which, such as NB-ARC domain genes, are the same as those enriched in SNPs and indels (Additional file 6).

### Genes with copy number variations

The annotation showed that 2,600 genes had 3,234 CNVs, and 32 genes had CNVs in all three sorghum lines. Some of these 32 genes encode proteins involved in basic biological functions, such as RNA polymerase beta subunit (*Sb04g009441, Sb04g009491, Sb02g017833*), NADH dehydrogenase subunit 6 (*Sb10g008595*), ribosomal protein S7 (*Sb05g020390*) and ribosomal protein S18 (*Sb02g032062*). One gene, *Sb04g035450*, was found to be lost in Keller and E-Tian, but gained extra copies in Ji2731. A blast revealed that it is a homologue of the glutamate-gated kainate-type ion channel receptor subunit gene *GluR5*. Gene family enrichment analysis showed that CNVs were statistically significantly (*P*-value < 0.001) enriched in genes encoding cellulose synthases, pectinesterases, GRAS transcription factors, and BTB/POZ and auxin responsive proteins, in addition to the DUF, leucine-rich repeat and the zinc knuckle proteins (Additional file 2).

### Genetic variation between sweet and grain sorghum

We speculated that some of the identified genetic variation might contribute to the phenotypic differentiation of sweet- and biofuel-associated traits and focused our analysis on SNPs, indels and PAVs in genic regions. For this, we used the gene set of the reference BTx623 genome as the control and identified all the shared variation within the two sweet sorghum lines and the variation between the reference genome and the Chinese local grain sorghum, respectively. Subsequently, the two sets of data were compared to remove those genes that have large-effect SNPs in the Chinese local grain sorghum, and the remaining gene set considered as those differentiating sweet and grain sorghum. We selected SNPs that are non-synonymous in the two sweet sorghum lines Keller and E-Tian but synonymous or even not present in the grain sorghum line Ji2731 (the reference BTx623 is a grain sorghum line). Similarly, for indels and PAVs, we selected those that were identified in both the Keller and E-Tian lines but not in Ji2731 and a gene was considered the putative differentiating gene when one indel was mapped to the coding region of the gene or the coding region of the gene was affected by a PAV.

**Figure 5:**
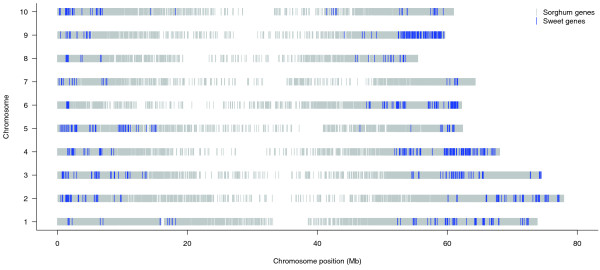
**Chromosomal locations of genes differentiating sweet and grain sorghum**. Genes with large-effect SNPs, indels and PAVs in the two sweet sorghum lines but devoid of these genetic variations in the grain sorghum were identified, considered as the sweet-associated genes, and mapped to the sorghum genome (see text for details). A 1-Mbp sliding window was used to define sweet-related regions on individual chromosomes, and only those windows containing more than three sweet-associated genes are shown. The overall gene distribution in the sorghum genome is shown by the grey bars as the background of every chromosome.

Figure 5 shows the chromosomal locations of these genes, most of which are scattered in the vicinity of subtelomeric regions, and obvious clusters of sweet-associated genes were found in genomic regions on chromosomes 4, 6 and 9. The selected SNP positions were mapped to 1,266 genes in this manner. A pathway enrichment analysis was performed for these genes, and as a result, ten Kyoto Encyclopedia of Genes and Genomes (KEGG) pathways, including starch and sucrose metabolism pathways and the lignin- and coumarine-biosynthesis associated phenylpropanoid biosynthesis pathways, were identified as statistically significantly (*P*-value < 0.1) enriched in the sweet-related gene sets (Table 4). In addition, 123 genes selected by indels and 53 genes selected by PAVs were identified. Among them, four genes were found to be in important gene families in previous studies. *Sb10g024663 *and *Sb03g032210 *were reported to be members of the P450 gene family, *Sb09g017540 *is a DREB transcription factor, and *Sb01g034050 *is in the expansin gene family. Owing to the limited grain and sweet sorghum genomes analyzed and the lack of a *de novo*-assembled complete sweet sorghum genome, the gene set reported here may not represent the whole spectrum of the genes differentiating sweet and grain sorghum and further in-depth study is required.

**Table 4:** Pathways statistically significantly (*P-*value < 0.1) enriched in the sweet-related gene set

KEGG ID	*P*-value	Pathway name
sbi00230	0.058	Purine metabolism
sbi00500	0.077	Starch and sucrose metabolism
sbi00240	0.017	Pyrimidine
sbi00592	0.017	alpha-Linolenic acid metabolism
sbi00780	0.070	Biotin metabolism
sbi00940	0.030	Phenylpropanoid biosynthesis
sbi04140	0.055	Regulation of autophagy
sbi04146	0.055	Peroxisome
sbi03430	0.005	Mismatch repair
sbi03018	0.087	RNA degradation

## Discussion

Rapid development of sequencing technologies and bioinformatic tools makes the complete genome sequencing of many species possible, which provides a starting point to unravel the tremendous genetic variation and diversity at the genome scale. Amongst several model organisms examined to date, such as human, mouse, *Arabidopsis*, rice, and maize, genome-wide patterns of genetic variation are able to be captured by sampling a relatively small number of genomes [14,20,50-52]. By resequencing two sweet and one grain sorghum inbred lines, we uncovered nearly two million SNPs and indels, along with large numbers of PAVs and CNVs. This is a first report on the genome-wide patterns of genetic variation in sorghum, which will be valuable for further genotype-phenotype studies and for molecular breeding of this important C_4 _model crop.

Our study shows that the proportions of genic SNPs identified as in coding regions, intronic regions, or UTRs are 42.3%, 50.2%, and 7.5%, respectively. Compared to *Arabidopsis *[19] and rice [14,20], the intronic regions of sorghum genes harbor more SNPs. This might be related to the increased size of the introns; the average intron size for *Arabidopsis *is 168 bp, and for rice it is 397 bp, but for sorghum it is 444 bp. Our results also demonstrate that, in sorghum, the proportions of large-effect SNPs resulting in premature stop codons, alteration of initiation methionine residues and disruption of splicing donor or acceptor sites are remarkably similar to what have been reported so far in *Arabidopsis *[19] and maize [22], but different from rice [20]. Furthermore, we found that 16 SNPs removed annotated stop codons and resulted in longer open reading frames, which is substantially smaller than the number (1,087) in maize.

It is known that transposon elements are abundant in sorghum as well as other cereal genomes [40,53]. As the genome annotation is not perfect, caution should be exercised with regard to the analysis of the effects of SNPs. Indeed, we found that the transposase genes, pseudogenes and low-confidence genes tended to have high non-synonymous-to-synonymous ratios in comparison with *bona fide *genes. This was reflected in the Pfam SNP annotations as well as in the analysis of so-called large-effect SNPs, which are predicted to disable gene functions. Most of the SNPs resided in receptor-like kinases, PPR repeats, disease resistant NB-ARC genes and other genes with multiple effects on stress responses. These genes also exhibited high non-synonymous-to-synonymous ratios, further supporting the notion from studies in other species that an arms race between plant-pathogen interactions results in diversification of the pathogen- or microbe-associated molecular pattern recognition receptors in plant genomes [54,55]. Significantly, the highest non-synonymous substitution ratios were found in X8 domain and glycoside hydrolase family 17 (glucan endo-1,3-beta-glucosidase) genes, which has not been reported in *Arabidopsis *[19], rice [20] or maize [22]. Current annotations show that limited low-confidence genes were included in these two Pfam gene families, although we cannot rule out the possibility that these genes are pseudogenes, or truncated because of the transposon elements. Further studies are required to validate whether they are related to specific biological processes in sorghum. However, the function of these genes in carbohydrate binding as well as in cell wall biosynthesis certainly provides clues to manipulating genes of interest for biofuel production.

In sorghum, the 14 gene families enriched with large-effect SNPs comprise genes encoding DUF proteins with unknown functions or include transponsons, which appear to be nonfunctional but may affect genetic variation at the genome scale. Furthermore, gene families involved in biotic and abiotic stress tolerance, which do not contain transposons, also harbor enriched large-effect SNPs. For instance, over-expression of lecithin:cholesterol acyltransferase can increase lipid metabolism and the fluidity of membranes and hence the resistance to heat and/or cold shock (United States Patent Application 20050150007), whereas chalcone synthase in flavonoid biosynthesis and stilbene synthases for phytoalexin biosynthesis play important roles in sorghum disease resistance [56,57]. None of these gene families were reported to be enriched with large-effect SNPs in *Arabidopsis*, rice or maize. This could be due to genome/species-specific diversity, or result from the prediction algorithms used. Alternatively, this may also be related to the limited sorghum lines used, which have diverse relationships.

This effort also uncovered substantial numbers of indels and PAVs in the sorghum genomes. Indels that are not multiples of 3 bp were particularly uncommon in coding regions but relatively common in non-coding regions. This implies that most frameshift mutations are harmful to sorghum survival. The spectrum of gene families affected by indels and PAVs was similar to that of large-effect SNPs. This implies that although the origins and scales of affected genome segments may differ, SNPs, indels and PAVs may share similar survival and distribution patterns, at least in terms of gene families affected. CNV studies in plants lag behind those in animal and human models. Recent studies in maize showed its potential contribution to the heterosis of this crop during domestication and disease responses [22,30,31]. CNVs also shaped the genome diversity of progeny of the immediate next generations in *Arabidopsis *[32]. In the sorghum genomes, CNVs were present in several thousand genes, and some of the commonly involved genes are involved in basic biological functions as well as sugar- and bioenergy-associated traits. How this variation is associated with phenotypic variation is a new direction of future research.

The resequenced sorghum lines contained two elite sweet sorghum lines and one local elite Chinese grain sorghum line. We were able to identify genetic variation in 1,442 genes differentiating sweet and grain sorghum. Some of these genes are involved in the starch and sucrose metabolism pathway and the lignin- and coumarine-biosynthesis-associated phenylpropanoid biosynthesis pathway, which are obvious candidates for sugar and biofuel production and deserve further study. Five genes in the starch and sucrose metabolism pathway were identified and are located on chromosomes 2, 6 and 9. In the phenylpropanoid biosynthesis pathway the cinnamyl-alcohol dehydrogenase gene (*Sb06g028240*, encoding EC 1.1.1.195) on chromosome 6 plays a central role in lignin biosynthesis. Previous genetic analyses have identified several quantitative trait loci controlling stem Brix content, grain yields, plant height and biomass on the same chromosomes [35,36,41,58]. However, due to the lack of the links between the genome physical map and the genetic linkage maps, it is hard to judge whether these genes and quantitative trait loci co-localize and further genetics and functional genomics studies are required to characterize the functions of these genes. Some of these gene families and pathways, may not be directly associated with sugar and biofuel traits, but rather reflect variation inherited from their different origins and/or caused by breeding selection. It is known that sweet sorghums are of polyphyletic origin, spreading from the kafir, caudatum, bicolor and other grain sorghum types [37,41]. Furthermore, using the BTx623 genome as a reference, the Chinese *kaoliang *line Ji2731 was found to harbor a lot more genetic variation than the other two lines (Additional file 6). Further genome-wide analysis with a panel of sweet and grain sorghum lines, close relatives of sorghum, as well as Chinese *kaoliang *is required to illustrate the complex relationships.

## Conclusions

We report here a whole genome map of SNPs, indels, PAVs and CNVs amongst elite sorghum lines, which can be used as a framework for future comparative and functional genomics. Sorghum is an important global crop, used for food, fodder, the production of alcoholic beverages, as well as biofuels. Genome-wide comparison studies with trait data of elite sorghum lines using the SNPs, indels, PAVs and CNVs discovered here will provide additional clues to the molecular basis of the remarkable traits of sweet sorghum and will provide a powerful source for association genetics and discovery of alleles, which can be combined to achieve crop improvement in the future.

## Materials and methods

### Plant materials and sequence data sets

Four sorghum (*S. bicolor*) accessions were used in this study. Btx623 is an elite grain sorghum line used for whole-genome sequencing by the Joint Genome Institute and for making several mapping populations [38]. Keller (GRIN access code PI 653617) is an elite sweet sorghum line developed by DM Broadhead at US Sugar Crops Field Station at Meridan, Mississippi in 1982 and has been grown globally and proven to have good performance across a range of environmental conditions [41]. E-Tian (literally meaning Russian Sweet in Chinese) was a line introduced to China in the early 1970s and is known to have high stem Brix content, while Ji2731 is a Chinese local grain sorghum well adapted to the northeastern part of China with good seedling establishment and a short growth period (Professor Shi-Jie Gao and Dr Wei-Bin Gu, personal communication; Archives of Crop Varieties in Jilin Province, 1988).

Seeds were imbibed and germinated at 25°C in darkness under standard glasshouse conditions for 4 days and 10-day-old etiolated seedlings were harvested for DNA isolation using the CTAB (Hexadecyl trimethyl-ammonium bromide) buffer method. Following quality assessment, genomic DNA was randomly fragmented using sonification and size-fractionated through electrophoresis and DNA fragments of the desired length were gel purified. Adapter ligation and DNA cluster preparation were performed and subjected to Solexa sequencing according to the supplier's protocol. The BTx623 reference genome sequences were downloaded from the Joint Genome Institute Phytozome website [46].

### Bioinformatics pipeline

#### SNP detection

We used a three-step procedure to detect high-quality SNPs. First, we calculated the likelihood of each accession's genotype using SOAPsnp [43]. Based on the alignment results, with consideration and analysis of data characteristics, sequencing quality and other experimental influences, the Bayesian model was applied to the actual data to calculate the probability of genotypes. The genotype with the highest probability was selected as the genotype of the sequencing individual at the specific locus and a quality value was designated accordingly to reveal the accuracy of the genotype. Second, using the consensus sequence, a polymorphic locus against the reference sequence was selected. Third, on the basis of the resequencing data of three accessions, sites with sufficient quality, called effective sites, were used for SNP determination. Sufficient quality was based on the following criteria: 3 ≤ depth ≤ 50, with depth calculated using data from each individual, and average mappable sites < 1.5. Candidate SNPs were those with sequencing depth of 3 to 50 for each accession and an average quality for the novel allele > 20. To exclude SNP calling errors caused by incorrect mapping or indels, we did not call two adjacent SNPs that were separated by < 5 bp. The remaining SNPs were defined as high quality SNPs. We performed SNP calling for each of the three accessions. These SNPs were used to calculate the whole genome SNP number and for further analysis. In SOAPsnp, a sum rank test was used to check the heterozygous sites of the called consensus. The read depth was used to filter the candidate SNPs and to obtain high accuracy heterozygous SNP sites. For a diploid genome, a site is considered heterozygous if each allele is supported by at least three reads.

#### SNP annotation

The localization of SNPs in coding regions, non-coding regions, start codons, stop codons and splice sites was based on annotation of gene models as provided by the *Sorghum bicolor *Genome Database [59]. Gene family annotation data of genes were also retrieved from this database.

#### Short indel detection

For short indel identification, mapped reads that met the pair-end requirements and contain alignment gaps in one end were necessary. We first mapped the paired-end reads to the reference sequence by allowing up to 10-bp gaps, and then merged these redundant pairs prior to looking for indels. Subsequently, gaps that were supported by at least three non-redundant paired-end reads were extracted. A potential indel was identified when the number of the un-gapped reads that crossed a potential indel was no more than twice that of the gapped reads. For quality control, the final list of indels included only those identified on both strands by paired-end reads.

#### Presence/absence variation detection

According to the principal of paired-end sequencing, one of the paired-end reads should normally be aligned onto the forward sequence, while the other should be aligned onto the reverse sequence. The distance between the two aligned positions at the reference should be in accordance with the insert size. Thus, two paired-end reads aligned to the genome should have a normal orientation and appropriate span. If the orientation or span of the two paired-end reads is different from expectation for the alignments results, the region might then have structure variation. The abnormal paired-end alignments are analyzed by clustering and compared with the types of structure variation previously defined. In this manner, PAVs could be detected, with support from at least three abnormal paired-end reads. In this study, PAVs that were supported by at least six paired-end reads were thought to be of high quality and selected as the final PAVs.

#### Copy number variation detection

We detected CNVs by the following steps: (i) DNA sequences were separated into fragments according to the depth of each base from the alignment results; (ii) we calculated the *P*-value for each fragment to estimate its probability to be a CNV; and (iii) fragments that passed the criteria (fragment length longer than 2 kb, *P*-value ≤0.35, mean depth less than 0.5 or more than 2.0) were kept as CNVs. The *P*-value was calculated as the probability of each observed depth (d) under the distribution of a simulated Poisson distributed data set whose expected value (E(d)) equals the observed mean depth. If d < E(d), the *P*-value = P(x ≤ d) × 2, else *P*-value = P(x ≥ d) × 2. The credibility of a CNV increases as the *P*-value becomes smaller.

#### Sweet-associated genes and pathway enrichment analysis

Using the BTx623 gene set as the reference, genes with non-synonymous SNPs identified in the Keller and E-Tian lines were selected as the candidate gene set. These genes were then analyzed to remove those that also contain non-synonymous SNPs in Ji2731 and the remaining genes were considered the sweet-associated genes. Sweet-associated genes were mapped to KEGG [60] sorghum pathway data and were examined for whether they are enriched in particular pathways based on the hypergeometric distribution test. Fisher's exact test was used to identify pathways significantly enriched (*P*-value < 0.1) with sweet-associated genes.

### Experimental validation

Two levels of data validation experiments were performed. For Sanger sequencing, primer sequences spanning genomic regions predicted to contain genetic variation were used to amplify genomic DNA templates from the three sorghum lines. For PCR reactions, each 10 μl reaction contained 50 ng of template DNA, 1.5 mM Mg^2+^, 1.5 mM dNTPs, 1.5 μM of each primer, 1 μl of 10 × PCR buffer, and 1.25 U of Taq DNA polymerase (Promega, China Branch, Beijing). The PCR conditions were 5 minutes at 95°C, followed by 30 cycles of 95°C/45 s, 58°C/45 s, and 72°C/90 s, ending with an extension of 72°C/5 minutes. The PCR products were subsequently analyzed using an ABI 3730 DNA analyzer.

### Data availability

The raw sequence data in the fastq format from this study were deposited in the NCBI Short Read Archive [61] under the accession number SRA046843. The annotated assemblies of *S. bicolor *genetic variations, including SNPs, indels, structural variations, and CNVs, as well as novel sequence contigs, are freely available from *GigaScience *[62]. The data have also been deposited in DDBJ/EMBL/ GenBank under the accession numbers AHAO00000000 (E-Tian cultivar); AHAP00000000 (Ji2731 cultivar); AHAQ00000000 (Keller cultivar). The versions described in this article are the first versions AHAO01000000, AHAP01000000 and AHAQ01000000.

## Abbreviations

Bp: base pairs; CNV: copy number variation; EST: expressed sequence tag; indel: insertion and deletion; KEGG: Kyoto Encyclopedia of Genes and Genomes; PAV: presence/absence variation; PPR: pentatricopeptide repeat; SNP: single nucleotide polymorphism; UTR: untranslated region.

## Competing interests

The authors declare that they have no competing interests.

## Authors' contributions

SR, CML, and HCJ conceived and designed the experiments. LYZ, XSG, BH, and LJS performed the experiments. XSG, BH, LJS, YP, SSD, TFL, SJ, SR, and HCJ analyzed the data; XSG, SR, CML, and HCJ contributed reagents/materials/analysis tools. XSG, BH, and HCJ wrote the paper.

## Supplementary Material

Additional file 1 **Tables S1 to S6, S8, and S11; legends for Figures S1 and S2**. Click here for file

Additional file 2 **Figure S1**. Click here for file

Additional file 3 **Table S7**. Click here for file

Additional file 4 **Table S9**. Click here for file

Additional file 5 **Table S10**. Click here for file

Additional file 6 **Figure S2**. Click here for file

## References

[B1] Rooney WL Blumenthal J Bean B Mullet JE Designing sorghum as a dedicated bioenergy feedstock. Biofuels Bioproducts Biorefining-Biofpr 2007 1 147 157 10.1002/bbb.15

[B2] Carpita NC McCann MC Maize and sorghum: genetic resources for bioenergy grasses. Trends Plant Sci 2008 13 415 420 10.1016/j.tplants.2008.06.002 18650120

[B3] Vermerris W Survey of genomics approaches to improve bioenergy traits in maize, sorghum and sugarcane. J Integr Plant Biol 2011 53 105 119 10.1111/j.1744-7909.2010.01020.x 21205186

[B4] Ashley-Koch A Yang Q Olney RS Sickle hemoglobin (HbS) allele and sickle cell disease: a HuGE review. Am J Epidemiol 2000 151 839 845 10791557 10.1093/oxfordjournals.aje.a010288

[B5] Kerem B Rommens JM Buchanan JA Markiewicz D Cox TK Chakravarti A Buchwald M Tsui LC Identification of the cystic fibrosis gene: genetic analysis. Science 1989 245 1073 1080 10.1126/science.2570460 2570460

[B6] Riordan JR Rommens JM Kerem B Alon N Rozmahel R Grzelczak Z Zielenski J Lok S Plavsic N Chou JL Identification of the cystic fibrosis gene: cloning and characterization of complementary DNA. Science 1989 245 1066 1073 10.1126/science.2475911 2475911

[B7] Rommens JM Iannuzzi MC Kerem B Drumm ML Melmer G Dean M Rozmahel R Cole JL Kennedy D Hidaka N Identification of the cystic fibrosis gene: chromosome walking and jumping. Science 1989 245 1059 1065 10.1126/science.2772657 2772657

[B8] Muller HJ Bar Duplication. Science 1936 83 528 530 17806465 10.1126/science.83.2161.528-a

[B9] Bridges CB The bar "gene" a duplication. Science 1936 83 210 211 10.1126/science.83.2148.210 17796454

[B10] Collard BC Mackill DJ Marker-assisted selection: an approach for precision plant breeding in the twenty-first century. Philos Trans R Soc Lond B Biol Sci 2008 363 557 572 10.1098/rstb.2007.2170 17715053 PMC2610170

[B11] Ganal MW Altmann T Roder MS SNP identification in crop plants. Curr Opin Plant Biol 2009 12 211 217 10.1016/j.pbi.2008.12.009 19186095

[B12] Langridge P Fleury D Making the most of 'omics' for crop breeding. Trends Biotechnol 2011 29 33 40 10.1016/j.tibtech.2010.09.006 21030098

[B13] Schmid KJ Sorensen TR Stracke R Torjek O Altmann T Mitchell-Olds T Weisshaar B Large-scale identification and analysis of genome-wide single-nucleotide polymorphisms for mapping in *Arabidopsis thaliana*. Genome Res 2003 13 1250 1257 10.1101/gr.728603 12799357 PMC403656

[B14] Feltus FA Wan J Schulze SR Estill JC Jiang N Paterson AH An SNP resource for rice genetics and breeding based on subspecies Indica and Japonica genome alignments. Genome Res 2004 14 1812 1819 10.1101/gr.2479404 15342564 PMC515328

[B15] Ossowski S Schneeberger K Clark RM Lanz C Warthmann N Weigel D Sequencing of natural strains of *Arabidopsis thaliana *with short reads. Genome Res 2008 18 2024 2033 10.1101/gr.080200.108 18818371 PMC2593571

[B16] Gore MA Chia JM Elshire RJ Sun Q Ersoz ES Hurwitz BL Peiffer JA McMullen MD Grills GS Ross-Ibarra J Ware DH Buckler ES A first-generation haplotype map of maize. Science 2009 326 1115 1117 10.1126/science.1177837 19965431

[B17] Huang X Wei X Sang T Zhao Q Feng Q Zhao Y Li C Zhu C Lu T Zhang Z Li M Fan D Guo Y Wang A Wang L Deng L Li W Lu Y Weng Q Liu K Huang T Zhou T Jing Y Li W Lin Z Buckler ES Qian Q Zhang QF Li J Han B Genome-wide association studies of 14 agronomic traits in rice landraces. Nat Genet 2010 42 961 967 10.1038/ng.695 20972439

[B18] Kump KL Bradbury PJ Wisser RJ Buckler ES Belcher AR Oropeza-Rosas MA Zwonitzer JC Kresovich S McMullen MD Ware D Balint-Kurti PJ Holland JB Genome-wide association study of quantitative resistance to southern leaf blight in the maize nested association mapping population. Nat Genet 2011 43 163 168 10.1038/ng.747 21217757

[B19] Clark RM Schweikert G Toomajian C Ossowski S Zeller G Shinn P Warthmann N Hu TT Fu G Hinds DA Chen H Frazer KA Huson DH Schölkopf B Nordborg M Rätsch G Ecker JR Weigel D Common sequence polymorphisms shaping genetic diversity in *Arabidopsis thaliana*. Science 2007 317 338 342 10.1126/science.1138632 17641193

[B20] McNally KL Childs KL Bohnert R Davidson RM Zhao K Ulat VJ Zeller G Clark RM Hoen DR Bureau TE Stokowski R Ballinger DG Frazer KA Cox DR Padhukasahasram B Bustamante CD Weigel D Mackill DJ Bruskiewich RM Rätsch G Buell CR Leung H Leach JE Genomewide SNP variation reveals relationships among landraces and modern varieties of rice. Proc Natl Acad Sci USA 2009 106 12273 12278 10.1073/pnas.0900992106 19597147 PMC2718348

[B21] Wang L Hao L Li X Hu S Ge S Yu J SNP deserts of Asian cultivated rice: genomic regions under domestication. J Evol Biol 2009 22 751 761 10.1111/j.1420-9101.2009.01698.x 19243488

[B22] Lai J Li R Xu X Jin W Xu M Zhao H Xiang Z Song W Ying K Zhang M Jiao Y Ni P Zhang J Li D Guo X Ye K Jian M Wang B Zheng H Liang H Zhang X Wang S Chen S Li J Fu Y Springer NM Yang H Wang J Dai J Schnable PS Wang J Genome-wide patterns of genetic variation among elite maize inbred lines. Nat Genet 2010 42 1027 1030 10.1038/ng.684 20972441

[B23] van Heerwaarden J Doebley J Briggs WH Glaubitz JC Goodman MM de Jesus Sanchez Gonzalez J Ross-Ibarra J Genetic signals of origin, spread, and introgression in a large sample of maize landraces. Proc Natl Acad Sci USA 2011 108 1088 1092 10.1073/pnas.1013011108 21189301 PMC3024656

[B24] Borevitz JO Liang D Plouffe D Chang HS Zhu T Weigel D Berry CC Winzeler E Chory J Large-scale identification of single-feature polymorphisms in complex genomes. Genome Res 2003 13 513 523 10.1101/gr.541303 12618383 PMC430246

[B25] Kumar R Qiu J Joshi T Valliyodan B Xu D Nguyen HT Single feature polymorphism discovery in rice. PLoS One 2007 2 e284 10.1371/journal.pone.0000284 17372626 PMC1808426

[B26] Bruce M Hess A Bai J Mauleon R Diaz MG Sugiyama N Bordeos A Wang GL Leung H Leach JE Detection of genomic deletions in rice using oligonucleotide microarrays. BMC Genomics 2009 10 129 10.1186/1471-2164-10-129 19320995 PMC2666768

[B27] Salathia N Lee HN Sangster TA Morneau K Landry CR Schellenberg K Behere AS Gunderson KL Cavalieri D Jander G Queitsch C Indel arrays: an affordable alternative for genotyping. Plant J 2007 51 727 737 10.1111/j.1365-313X.2007.03194.x 17645438

[B28] Fu H Dooner HK Intraspecific violation of genetic colinearity and its implications in maize. Proc Natl Acad Sci USA 2002 99 9573 9578 12060715 10.1073/pnas.132259199 PMC123182

[B29] Brunner S Fengler K Morgante M Tingey S Rafalski A Evolution of DNA sequence nonhomologies among maize inbreds. Plant Cell 2005 17 343 360 10.1105/tpc.104.025627 15659640 PMC548811

[B30] Springer NM Ying K Fu Y Ji T Yeh CT Jia Y Wu W Richmond T Kitzman J Rosenbaum H Iniguez AL Barbazuk WB Jeddeloh JA Nettleton D Schnable PS Maize inbreds exhibit high levels of copy number variation (CNV) and presence/absence variation (PAV) in genome content. PLoS Genet 2009 5 e1000734 10.1371/journal.pgen.1000734 19956538 PMC2780416

[B31] Belo A Beatty MK Hondred D Fengler KA Li B Rafalski A Allelic genome structural variations in maize detected by array comparative genome hybridization. Theor Appl Genet 2009 120 355 367 19756477 10.1007/s00122-009-1128-9

[B32] DeBolt S Copy number variation shapes genome diversity in Arabidopsis over immediate family generational scales. Genome Biol Evol 2010 2 441 453 10.1093/gbe/evq033 20624746 PMC2997553

[B33] Harlan JR deWet JWJ A simplified classification of sorghum. Crop Sci 1972 12 172 176 10.2135/cropsci1972.0011183X001200020005x

[B34] Vietor DM Miller FR Assimilation, partitioning, and nonstructural carbohydrate in sweet compared with grain sorghum. Crop Sci 1990 30 1109 1115 10.2135/cropsci1990.0011183X003000050030x

[B35] Murray SC Sharma A Rooney WL Klein PE Mullet JE Mitchell SE Kresovich S Genetic improvement of sorghum as a biofuel feedstock: I. QTL for stem sugar and grain nonstructural carbohydrates. Crop Sci 2008 48 2165 2179 10.2135/cropsci2008.01.0016

[B36] Murray SC Rooney WL Mitchell SE Sharma A Klein PE Mullet JE Kresovich S Genetic improvement of sorghum as a biofuel feedstock: II. QTL for stem and leaf structural carbohydrates. Crop Sci 2008 48 2180 2193 10.2135/cropsci2008.01.0068

[B37] Ritter KB McIntyre CL Godwin ID Jordan DR Chapman SC An assessment of the genetic relationship between sweet and grain sorghums, within Sorghum bicolor ssp bicolor (L.) Moench, using AFLP markers. Euphytica 2007 157 161 176 10.1007/s10681-007-9408-4

[B38] Paterson AH Genomics of sorghum. Int J Plant Genomics 2008 2008 362451 18483564 10.1155/2008/362451 PMC2375965

[B39] Draye X Lin YR Qian XY Bowers JE Burow GB Morrell PL Peterson DG Presting GG Ren SX Wing RA Paterson AH Toward integration of comparative genetic, physical, diversity, and cytomolecular maps for grasses and grains, using the sorghum genome as a foundation. Plant Physiol 2001 125 1325 1341 10.1104/pp.125.3.1325 11244113 PMC65612

[B40] Paterson AH Bowers JE Bruggmann R Dubchak I Grimwood J Gundlach H Haberer G Hellsten U Mitros T Poliakov A Schmutz J Spannagl M Tang H Wang X Wicker T Bharti AK Chapman J Feltus FA Gowik U Grigoriev IV Lyons E Maher CA Martis M Narechania A Otillar RP Penning BW Salamov AA Wang Y Zhang L Carpita NC The Sorghum bicolor genome and the diversification of grasses. Nature 2009 457 551 556 10.1038/nature07723 19189423

[B41] Wang ML Zhu C Barkley NA Chen Z Erpelding JE Murray SC Tuinstra MR Tesso T Pederson GA Yu J Genetic diversity and population structure analysis of accessions in the US historic sweet sorghum collection. Theor Appl Genet 2009 120 13 23 10.1007/s00122-009-1155-6 19760215

[B42] SOAP. http://soap.genomics.org.cn

[B43] Li R Li Y Fang X Yang H Wang J Kristiansen K SNP detection for massively parallel whole-genome resequencing. Genome Res 2009 19 1124 1132 10.1101/gr.088013.108 19420381 PMC2694485

[B44] Yoon S Xuan Z Makarov V Ye K Sebat J Sensitive and accurate detection of copy number variants using read depth of coverage. Genome Res 2009 19 1586 1592 10.1101/gr.092981.109 19657104 PMC2752127

[B45] Phytozome Sorghum bicolor (Cereal grass). http://www.phytozome.net/sorghum

[B46] MaizeGDB. http://www.maizegdb.org

[B47] Rudrabhatla P Reddy MM Rajasekharan R Genome-wide analysis and experimentation of plant serine/threonine/tyrosine-specific protein kinases. Plant Mol Biol 2006 60 293 319 10.1007/s11103-005-4109-7 16429265

[B48] Thomas JH Adaptive evolution in two large families of ubiquitin-ligase adapters in nematodes and plants. Genome Res 2006 16 1017 1030 10.1101/gr.5089806 16825662 PMC1524861

[B49] Hardie DG Plant protein serine/threonine kinases: classification and functions. Annu Rev Plant Physiol Plant Mol Biol 1999 50 97 131 10.1146/annurev.arplant.50.1.97 15012205

[B50] Frazer KA Eskin E Kang HM Bogue MA Hinds DA Beilharz EJ Gupta RV Montgomery J Morenzoni MM Nilsen GB Pethiyagoda CL Stuve LL Johnson FM Daly MJ Wade CM Cox DR A sequence-based variation map of 8.27 million SNPs in inbred mouse strains. Nature 2007 448 1050 1053 10.1038/nature06067 17660834

[B51] Kim S Plagnol V Hu TT Toomajian C Clark RM Ossowski S Ecker JR Weigel D Nordborg M Recombination and linkage disequilibrium in *Arabidopsis thaliana*. Nat Genet 2007 39 1151 1155 10.1038/ng2115 17676040

[B52] Hinds DA Stuve LL Nilsen GB Halperin E Eskin E Ballinger DG Frazer KA Cox DR Whole-genome patterns of common DNA variation in three human populations. Science 2005 307 1072 1079 10.1126/science.1105436 15718463

[B53] Schnable PS Ware D Fulton RS Stein JC Wei F Pasternak S Liang C Zhang J Fulton L Graves TA Minx P Reily AD Courtney L Kruchowski SS Tomlinson C Strong C Delehaunty K Fronick C Courtney B Rock SM Belter E Du F Kim K Abbott RM Cotton M Levy A Marchetto P Ochoa K Jackson SM Gillam B The B73 maize genome: complexity, diversity, and dynamics. Science 2009 326 1112 1115 10.1126/science.1178534 19965430

[B54] Stahl EA Dwyer G Mauricio R Kreitman M Bergelson J Dynamics of disease resistance polymorphism at the Rpm1 locus of Arabidopsis. Nature 1999 400 667 671 10.1038/23260 10458161

[B55] McDowell JM Dhandaydham M Long TA Aarts MG Goff S Holub EB Dangl JL Intragenic recombination and diversifying selection contribute to the evolution of downy mildew resistance at the RPP8 locus of Arabidopsis. Plant Cell 1998 10 1861 1874 9811794 10.1105/tpc.10.11.1861 PMC143965

[B56] Winkel-Shirley B Flavonoid biosynthesis. A colorful model for genetics, biochemistry, cell biology, and biotechnology. Plant Physiol 2001 126 485 493 10.1104/pp.126.2.485 11402179 PMC1540115

[B57] Yu CK Springob K Schmidt J Nicholson RL Chu IK Yip WK Lo C A stilbene synthase gene (SbSTS1) is involved in host and nonhost defense responses in sorghum. Plant Physiol 2005 138 393 401 10.1104/pp.105.059337 15821144 PMC1104192

[B58] Shiringani AL Frisch M Friedt W Genetic mapping of QTLs for sugar-related traits in a RIL population of Sorghum bicolor L. Moench. Theor Appl Genet 2010 121 323 336 10.1007/s00122-010-1312-y 20229249

[B59] *Sorghum bicolor *genome. http://www.plantgdb.org/SbGDB/

[B60] KEGG: Kyoto Encyclopedia of Genes and Genomes. http://www.genome.jp/kegg/

[B61] Sequence Read Archive. http://www.ncbi.nlm.nih.gov/sra

[B62] Zheng LY Guo XS He B Sun LJ Peng Y Dong SS Liu TF Jiang S Ramachandran S Liu CM Jing H-C Genome data from sweet and grain sorghum (*Sorghum bicolour*). GigaScience 2011 http://dx.doi.org/10.5524/100012 10.1186/gb-2011-12-11-r114 PMC3334600 22104744

