# Supplementary material for: Genome-wide patterns of genetic variation in sweet and grain sorghum (Sorghum bicolor)
Source: Genome Biol. 2011 Nov 21;12(11):R114. doi: 10.1186/gb-2011-12-11-r114 (PMC3334600; doi:10.1186/gb-2011-12-11-r114)
Supplement: Additional file 1 — Tables S1 to S6, S8, and S11; legends for Figures S1 and S2. [file gb-2011-12-11-r114-S1.DOC]

**Additional File1**

**Figure S1. Summary of SNPs and Indels in three sorghum accessions**

(**A**) Overlap of SNPs in genome.

(**B**) Overlap of SNPs in CDS.

(**C**) Overlap of genes that contain SNPs.

(**D**) Overlap of indels in genome.

(**E**) Overlap of indels in CDS.

(**F**) Overlap of genes that contain Indels

**Figure S2.** A phylogenetic tree for the sequenced sorghums with rice as an outgroup. In total, SNPs from 6850 gene pairs in these sorghum lines were used to reconstruct the phylogenetic tree.

**Table S1.** **Summary of re-sequencing coverage**

| **Sample** | **Total reads(M)** | **Mapped reads (G)** | **Total bases (G)** | **Mapped bases (G)** | **Depth** | **Coverage(%)** | **Unique(%)** | **Mismatch(%)** |
| --- | --- | --- | --- | --- | --- | --- | --- | --- |
|
| **Ji2731** | 216.13 | 193.61 | 9.51 | 8.52 | 12.71 | 89.63 | 63.06% | 0.44% |
| **Keller** | 199.84 | 186.08 | 8.79 | 8.19 | 11.76 | 91.44 | 67.08% | 0.38% |
| **E-Tian** | 204.75 | 187.81 | 9.01 | 8.26 | 12.04 | 87.97 | 64.58% | 0.37% |

**Table S2. Distribution of SNPs in sorghum genome**

| **Sample** | **Total** | **UTR** | | **CDS** | | **Intron** | **InterGene** |
| --- | --- | --- | --- | --- | --- | --- | --- |
|  |  | **5’-UTR** | **3’-UTR** | **Syn** | **Non-syn** |  |  |
| **Ji2731** | 555,547 | 2,484 | 5,935 | 21,041 | 27,660 | 51,541 | 446,886 |
| **Keller** | 389,537 | 1,060 | 4,635 | 11,886 | 16,929 | 46,382 | 308,640 |
| **E-Tian** | 427,368 | 1,964 | 4,368 | 16,110 | 21,053 | 36,651 | 347,222 |

**Table S3. Heterozygosity of SNPs in sorghum genome**

| **Sample** | **CDS** | | | **Genome** | | |
| --- | --- | --- | --- | --- | --- | --- |
|  | **Hetero** | **Homo** | **Heterozygosity** | **Hetero** | **Homo** | **Heterozygosity** |
| **Ji2731** | 6471 | 42240 | 0.13 | 110503 | 472220 | 0.19 |
| **Keller** | 5042 | 23783 | 0.17 | 87299 | 318365 | 0.22 |
| **E-Tian** | 5408 | 31765 | 0.15 | 101006 | 347973 | 0.22 |
|  |  |  |  |  |  |  |

**Table S4. Distribution of Indels in sorghum genome**

| **Sample** | **Total** | **UTR** | | **CDS** | **Intron** | **InterGene** |
| --- | --- | --- | --- | --- | --- | --- |
|  |  | **5'-UTR** | **3'-UTR** |  |  |  |
| **Ji2731** | 50,402 | 578 | 1,324 | 1,241 | 9,244 | 38,015 |
| **Keller** | 41,395 | 302 | 1,005 | 798 | 8,383 | 30,907 |
| **E-Tian** | 33,833 | 446 | 860 | 922 | 5,862 | 25,743 |

**Table S5. Number of PAVs in sorghum genome**

| **Sample** | **Total** | **Presence** | **Absence** |
| --- | --- | --- | --- |
| **Ji2731** | 9,080 | 153 | 8,927 |
| **Keller** | 3,582 | 777 | 2,805 |
| **E-Tian** | 3,826 | 29 | 3,797 |

**Table S6.** **Number of genes affected by Indels and SVs (PAVs and CNVs)**

| **Sample** | **Indel** | **SV** | **Overlap** | **Total** |
| --- | --- | --- | --- | --- |
| **Ji2731** | 1,132 | 937 | 45 | 2,024 |
| **Keller** | 749 | 381 | 13 | 1,117 |
| **E-Tian** | 839 | 626 | 28 | 1,437 |

**Table S8. Number of genes discovered by reconstruction of the unmapped reads which are absent in the Btx623 genome. The corresponding number of homologues in *Arabidopsis,* maize and rice are shown.**

| **Sample** | **Gene** | ***Arabidopsis* homologue** | **Maize homologue** | **Rice homologue** | **Total homologue** |
| --- | --- | --- | --- | --- | --- |
| **Ji2731** | 35 | 3 | 3 | 1 | 4 |
| **Keller** | 22 | 21 | 21 | 2 | 21 |
| **E-Tian** | 16 | 4 | 7 | 1 | 8 |

| Auxin responsive protein  Auxin responsive protein | BTB/POZ domain | Cellulose synthase | Cytochrome P450 | Dimerisation domain | D-mannose binding lectin | Domain of unknown function (DUF1719) | Endonuclease/Exonuclease/phosphatase family | FAR1 family | F-box domain | FNIP Repeat | GRAS family transcription factor | Integrase core domain | Legume lectin domain | Leucine Rich Repeat | NB-ARC domain | PAN domain | PAN-like domain | Pectinesterase | Protein kinase domain | Protein of unknown function (DUF1618) | Protein of unknown function (DUF295) | Protein of unknown function, DUF594 | Protein tyrosine kinase | Reverse transcriptase (RNA-dependent DNA polymerase) | S-locus glycoprotein family | SWIM zinc finger | Zinc finger, C2H2 type | Zinc knuckle | **PFAM gene family** | | **Table S11.** G**ene families statistically significantly (*P* value <0.001) enriched with non-frame-shift Indels (3bp,6bp,9bp), frame shift Indels, 1-10bp Indels, PAVs and CNVs** |
| --- | --- | --- | --- | --- | --- | --- | --- | --- | --- | --- | --- | --- | --- | --- | --- | --- | --- | --- | --- | --- | --- | --- | --- | --- | --- | --- | --- | --- | --- | --- | --- |
| / | / | / | 1.58E-06 | / | 4.08E-04 | / | / | / | / | / | / | / | / | / | 2.58E-05 | / | 3.76E-04 | / | 6.95E-04 | 4.49E-04 | / | 1.30E-11 | 2.34E-04 | / | 1.71E-04 | / | 4.92E-04 | / | **P value** | **Non-frame-shift indel** |
| / | / | / | 30 | / | 11 | / | / | / | / | / | / | / | / | / | 21 | / | 9 | / | 60 | 7 | / | 18 | 54 | / | 9 | / | 13 | / | **Affected genes** |
| / | / | / | / | / | / | 8.83E-06 | / | / | 1.75E-09 | / | / | / | / | 3.55E-08 | 1.06E-08 | / | / | / | / | 2.68E-04 | / | 1.29E-06 | / | / | / | / | / | / | **P value** | **Frame-shift indel** |
| / | / | / | / | / | / | 7 | / | / | 40 | / | / | / | / | 46 | 23 | / | / | / | / | 7 | / | 11 | / | / | / | / | / | / | **Affected genes** |
| / | / | / | 8.36E-05 | 3.43E-04 | 5.98E-06 | 3.44E-05 | / | / | 3.40E-07 | / | / | / | / | 2.17E-05 | 6.56E-11 | 6.26E-04 | 1.50E-05 | / | 5.21E-05 | 7.87E-05 | / | 2.48E-15 | 5.95E-06 | / | 4.21E-06 | / | / | / | **P value** | **Indel** |
| / | / | / | 37 | 10 | 18 | 9 | / | / | 61 | / | / | / | / | 71 | 40 | 9 | 14 | / | 97 | 10 | / | 26 | 88 | / | 14 | / | / | / | **Affected genes** |
| / | / | / | / | / | / | 1.21E-05 | 3.08E-04 | 4.75E-05 |  | 9.43E-04 | / | 3.90E-05 | / | / | 2.59E-04 | / | / | / | / | / | / | / | / | 5.50E-05 | / | 3.44E-05 | / | 2.58E-04 | **P value** | **PAV** |
| / | / | / | / | / | / | 8 | 11 | 13 | / | 4 | / | 9 | / | / | 20 | / | / | / | / | / | / | / | / | 15 | / | 18 | / | 26 | **Affected genes** |
| 3.31E-07 | 3.61E-04 | 2.03E-04 | / | / | / | / | / | / | / | / | 1.91E-04 |  | 4.08E-05 | 1.75E-04 | / | / | / | 9.99E-04 |  | 3.01E-04 | 1.13E-05 | 4.38E-15 | / | 2.08E-07 | / | / | / | 1.29E-05 | **P value** | **CNV** |
| 18 | 20 | 9 | / | / | / | / | / | / | / | / | 14 |  | 13 | 74 | / | / | / | 9 |  | 11 | 19 | 27 | / | 25 | / | / | / | 42 | **Affected genes** |  |
