# Supplementary figures and images for: Genome-wide patterns of genetic variation in sweet and grain sorghum (Sorghum bicolor)
Source: Genome Biol. 2011 Nov 21;12(11):R114. doi: 10.1186/gb-2011-12-11-r114 (PMC3334600; doi:10.1186/gb-2011-12-11-r114)

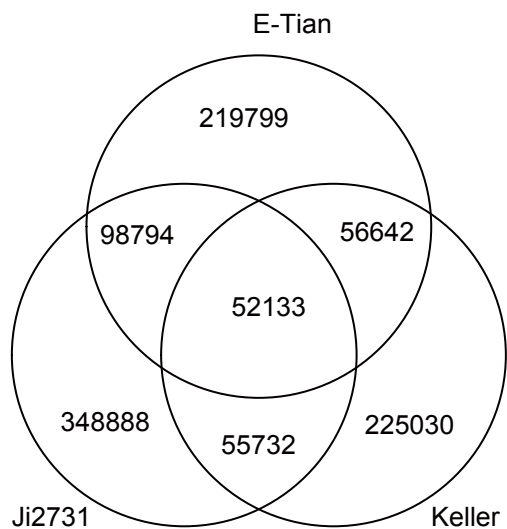

A

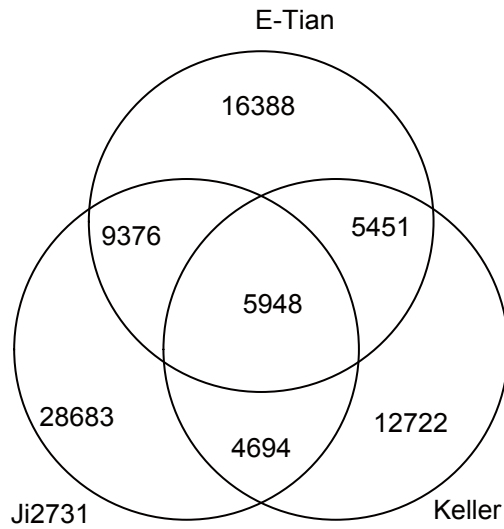

B

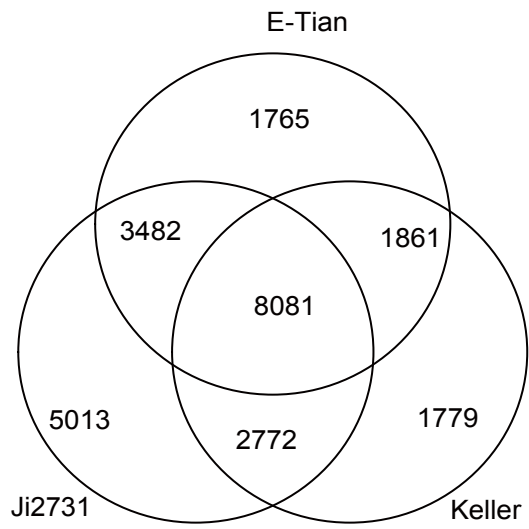

C

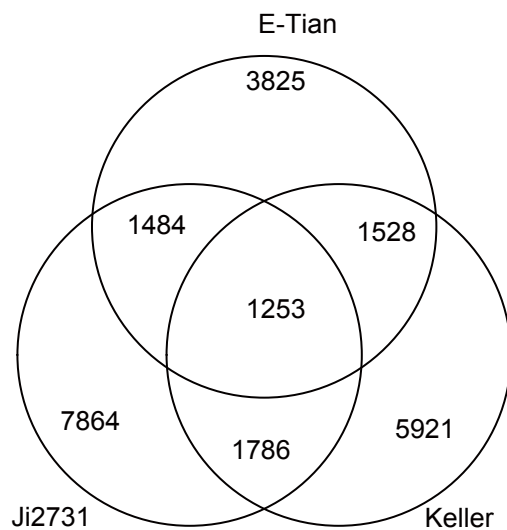

D

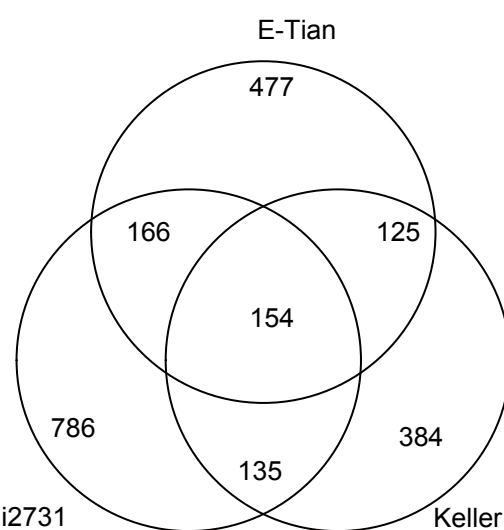

E

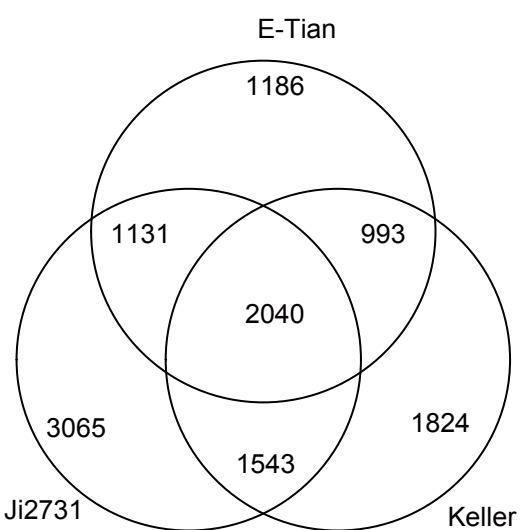

F

Supplement: Additional file 2 — Figure S1. [file gb-2011-12-11-r114-S2.PDF]

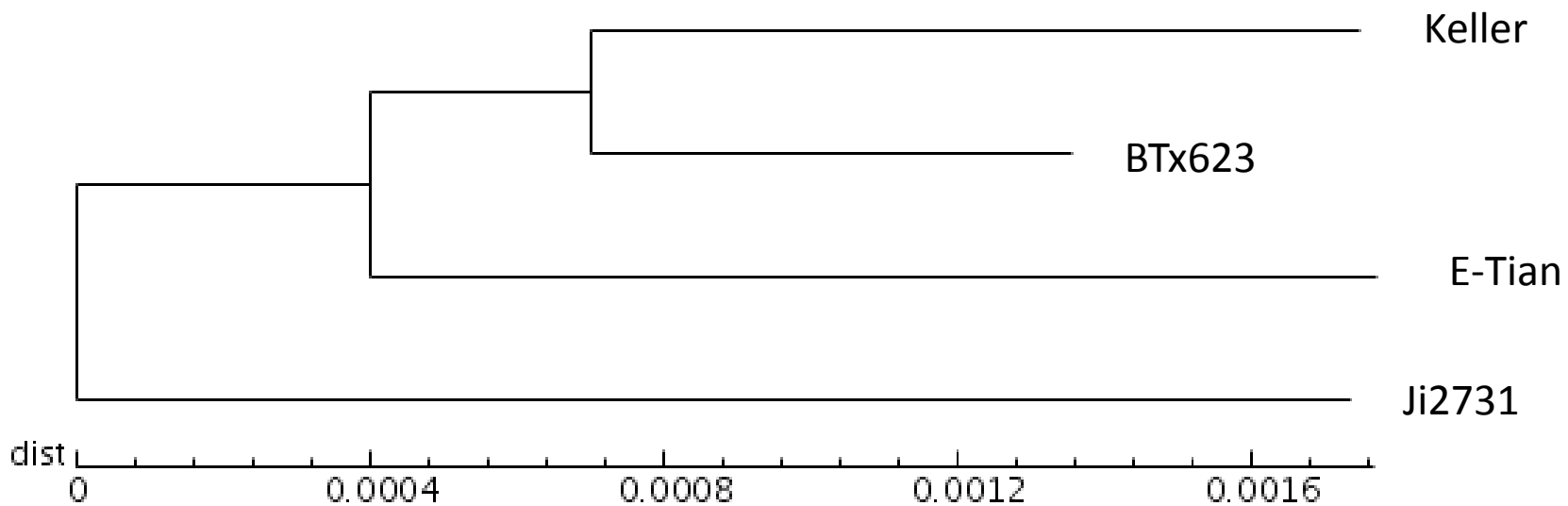

Supplement: Additional file 6 — Figure S2. [file gb-2011-12-11-r114-S6.PDF]
